# Clinical characteristics and outcomes of patients with COVID-19 admitted to the intensive care unit during the first and second waves of the pandemic in Brazil: a single-center retrospective cohort study

**DOI:** 10.31744/einstein_journal/2023AO0233

**Published:** 2023-07-06

**Authors:** Thiago Domingos Corrêa, Thais Dias Midega, Ricardo Luiz Cordioli, Carmen Silvia Valente Barbas, Roberto Rabello, Bruno Caldin da Silva, Moacyr Silva, Ricardo Kenji Nawa, Fabrício Rodrigues Torres de Carvalho, Gustavo Faissol Janot de Matos, Neide Marcela Lucinio, Rodrigo Dias Rodrigues, Raquel Afonso Caserta Eid, Bruno de Arruda Bravim, Adriano José Pereira, Bento Fortunato Cardoso dos Santos, João Renato Rebello Pinho, Andreia Pardini, Vanessa Damazio Teich, Claudia Regina Laselva, Miguel Cendoroglo, Sidney Klajner, Leonardo José Rolim Ferraz

**Affiliations:** 1 Hospital Israelita Albert Einstein São Paulo SP Brazil Hospital Israelita Albert Einstein, São Paulo, SP, Brazil.

**Keywords:** Coronavirus infections, COVID-19, SARS-CoV-2, Respiration, artificial, Noninvasive ventilation, Extracorporeal membrane oxygenation, Critical care outcomes, Mortality, Intensive care units

## Abstract

**Objective:**

To describe and compare the clinical characteristics and outcomes of patients admitted to intensive care units during the first and second waves of the COVID-19 pandemic.

**Methods:**

In this retrospective single-center cohort study, data were retrieved from the Epimed Monitor System; all adult patients admitted to the intensive care unit between March 4, 2020, and October 1, 2021, were included in the study. We compared the clinical characteristics and outcomes of patients admitted to the intensive care unit of a quaternary private hospital in São Paulo, Brazil, during the first (May 1, 2020, to August 31, 2020) and second (March 1, 2021, to June 30, 2021) waves of the COVID-19 pandemic.

**Results:**

In total, 1,427 patients with COVID-19 were admitted to the intensive care unit during the first (421 patients) and second (1,006 patients) waves. Compared with the first wave group [median (IQR)], the second wave group was younger [57 (46-70) *versus* 67 (52-80) years; p<0.001], had a lower SAPS 3 Score [45 (42-52) *versus* 49 (43-57); p<0.001], lower SOFA Score on intensive care unit admission [3 (1-6) *versus* 4 (2-6); p=0.018], lower Charlson Comorbidity Index [0 (0-1) *versus* 1 (0-2); p<0.001], and were less frequently frail (10.4% *versus* 18.1%; p<0.001). The second wave group used more noninvasive ventilation (81.3% *versus* 53.4%; p<0.001) and high-flow nasal cannula (63.2% *versus* 23.0%; p<0.001) during their intensive care unit stay. The intensive care unit (11.3% *versus* 10.5%; p=0.696) and in-hospital mortality (12.3% *versus* 12.1%; p=0.998) rates did not differ between both waves.

**Conclusion:**

In the first and second waves, patients with severe COVID-19 exhibited similar mortality rates and need for invasive organ support, despite the second wave group being younger and less severely ill at the time of intensive care unit admission.

**Figure:**
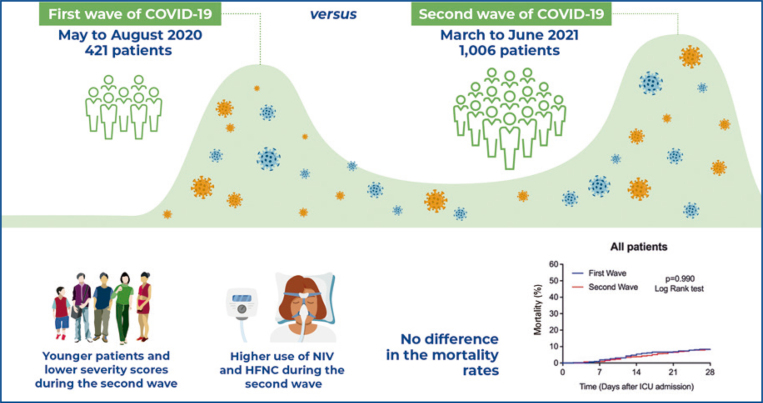

## INTRODUCTION

Coronavirus disease 2019 (COVID-19) has become a major public health concern, with almost half a billion cases diagnosed and over six million deaths worldwide.^(1)^ During this pandemic, waves of increased numbers of patients newly diagnosed with severe acute respiratory syndrome coronavirus 2 (SARS-CoV-2) infection have been reported worldwide, with varying degrees of disease severity and pressure on healthcare systems.^(2)^

Since the first SARS-CoV-2 infection was confirmed in São Paulo, Brazil,^(3)^ over 31 million cases and 660 thousand deaths due to COVID-19 have been registered.^(1)^ Brazil’s first wave of COVID-19 occurred between March and November 2020, and the most prevalent SARS-CoV-2 variants were B.1.1.28 and B.1.1.33.^(4)^The second wave occurred between February and October 2021, when the most prevalent SARS-CoV-2 variants were P.2 and P.1 (Gamma).^(4)^During both waves, an abrupt increase in new COVID-19 cases across different Brazilian regions imposed enormous pressure on the healthcare system, which had been under strain since the beginning of the pandemic.^(5)^

Studies comparing the epidemiological characteristics and outcomes of both waves in different countries have reported conflicting results.^(4-10)^ While some authors reported lower mortality rates during the second wave,^(6,8)^ others found no significant differences in mortality^(9,10)^ or reported poorer clinical outcomes, such as increased mortality among younger age groups and an increased proportion of patients requiring mechanical ventilation.^(5,7)^ A cross-sectional study of a large nationwide database of hospitalized patients in Brazil showed that, compared with the first wave, the second wave was characterized by increased demand for intensive care unit (ICU) admissions, increased use of noninvasive ventilation (NIV), invasive mechanical ventilation (MV), and increased hospital mortality.^(4)^

The clinical characteristics and outcomes of patients with COVID-19 in Brazil vary significantly across the country, mainly because of social and economic disparities and different levels of access to the health system.^(4,11)^ Studies on the epidemiological characteristics and outcomes of patients with severe COVID-19 admitted to the ICU in private hospitals in Brazil during the first two waves of the pandemic are limited.

## OBJECTIVE

To describe and compare the epidemiological and clinical characteristics, resource use, and outcomes of patients with COVID-19 admitted to the intensive care unit during the first and second waves of the COVID-19 pandemic in São Paulo, Brazil.

## METHODS

### Study design

This was a single-center retrospective cohort study. The study was approved by the local ethics committee of *Hospital Israelita Albert Einstein*; the need for informed consent was waived (CAAE: 30797520.6.0000.0071; # 4.562.815). This study was conducted in accordance with the Strengthening the Reporting of Observational Studies in Epidemiology (STROBE) Statement.^(12)^

### Setting

This study was conducted in a private quaternary care hospital in São Paulo, Brazil. The *Hospital Israelita Albert Einstein* has 706 beds. Of these, 37 were open medical-surgical adult ICU beds, and 81 were adult step-down unit beds. The total ICU operational capacity designated to support patients with severe COVID-19 requiring intensive care increased during the first and second waves, reaching 81 and 159 ICU beds, respectively.

### Study participants

Consecutive adult (≥18 years) patients admitted to the ICU from March 4, 2020, to October 1, 2021, and diagnosed with COVID-19 were eligible for inclusion in this study. Laboratory confirmation of SARS-CoV-2 infection was based on a positive reverse transcription polymerase chain reaction (RT-PCR) assay.^(13)^

### Patient management

The criteria for ICU admission and institutional protocol for severe SARS-CoV-2 infection management have been published elsewhere.^(14,15)^

### Data collection and study variables

All study data were retrieved from the Epimed Monitor System^®^ (Epimed Solutions, Rio de Janeiro, Brazil), an electronic structured case report form in which trained ICU case managers entered patient data prospectively.^(16)^ Collected variables included demographics, comorbidities, Simplified Acute Physiology Score (SAPS 3 Score)^(17)^ at ICU admission, Sequential Organ Failure Assessment (SOFA) Score^(18)^ at ICU admission, Charlson Comorbidity Index,^(19)^ Modified Frailty Index (MFI),^(20,21)^ resource use and organ support [vasopressors, NIV, high flow nasal cannula (HFNC), MV, renal replacement therapy (RRT) and extracorporeal membrane oxygenation (ECMO)] during ICU stay, destination at hospital discharge, ICU and hospital length of stay (LOS), and ICU and in-hospital mortality.

### Definitions

We defined the first wave period as the time from May 1, 2020, to August 31, 2020 (first wave group) and the second wave from March 1, 2021, to June 30, 2021 (second wave group). These two periods correspond to four consecutive months in 2020 and 2021, respectively, with the highest number of patients with COVID-19 admitted to the ICU.

### Statistical analysis

Categorical variables were presented as absolute and relative frequencies. Continuous variables were presented as medians with interquartile ranges (IQR). Normality was assessed using the Kolmogorov-Smirnov test.

The first- and second-wave groups were compared. Categorical variables were compared with the χ^2^ test or Fisher’s exact test as appropriate. Continuous variables were compared using an independent Student’s *t* test or the Mann-Whitney U test in cases of non-normal distribution. Mortality on day 28 of the pooled patients and mortality stratified according to the use of mechanical ventilation, RRT, and ECMO were analyzed using the Kaplan-Meier method. Patients discharged from the hospital before day 28 were considered alive on day 28.

Two-tailed tests were used, and statistical significance was set at p<0.05. All analyses were performed using the IBM (SPSS) Statistics for Macintosh, version 27 (IBM Corp., Armonk, NY, USA), and GraphPad Prism version 9.3.0 (GraphPad Software Inc., San Diego, CA, USA) was used for graph plotting.

## RESULTS

From March 4, 2020, to October 1, 2021, 2,566 patients with COVID-19 were admitted to the ICU. Of them, 1,427 were admitted during the first (421 patients) and second (1,006 patients) COVID-19 waves.

### Patient characteristics

The baseline characteristics of the patients admitted during the first and second waves are shown in table 1. Compared with the first wave group, the second wave group was younger [57 (46-70) *versus* 67 (52-80) years; p<0.001]. More often, they were men (69.9% *versus* 63.9%; p=0.032), had a lower SAPS 3 Score [45 (42-52) *versus* 49 (43-57); p<0.001], a lower SOFA Score [3 (1-6) *versus* 4 (2-6); p=0.018], lower Charlson Comorbidity Index [0 (0-1) *versus* 1 (0-2); p<0.001], were less frequently frail (10.4% *versus* 18.1%; p<0.001), and had less frequently congestive heart failure (3.5% *versus* 7.2%; p=0.012).

**Table 1:** Baseline characteristics of studied patients

Characteristics	All n=1,427	First wave n=421	Second wave n=1,006	p value*
Age, years (median, IQR)	59 (47-73)	67 (52-80)	57 (46-70)	<0.001^#^
Men, n/total n (%)	972/1,427 (68.1)	269/421 (63.9)	703/1,006 (69.9)	0.032^&^
SAPS 3 Score (median, IQR)	46 (42-54)	49 (43-57)	45 (42-52)	<0.001^#^
SOFA Score (median, IQR)^$^	3 (1-6)	4 (2-6)	3 (1-6)	0.018^#^
CCI (median, IQR)	0 (0-1)	1 (0-2)	0 (0-1)	<0.001^#^
MFI, points (median, IQR)^‡^	1 (0-2)	1 (0-2)	1 (0-2)	<0.001^#^
Non-frail	612/1,416 (43.2)	153/421 (36.3)	459/995 (46.1)	<0.001^&^
Pre-frail	625/1,416 (44.1)	192/421 (45.6)	433/995 (43.5)	
Frail	179/1,416 (12.6)	76/421 (18.1)	103/995 (10.4)	
Underlying disease, n/total n (%)				
Systemic hypertension	623/1,083 (57.5)	195/335 (58.2)	428/748 (57.2)	0.812^&^
*Diabetes mellitus*	328/1,083 (30.3)	113/335 (33.7)	215/748 (28.7)	0.114^&^
Asthma	68/1,083 (6.3)	23/335 (6.9)	45/748 (6.0)	0.691^&^
Cancer	72/1,083 (6.6)	23/335 (6.9)	49/748 (6.6)	0.952^&^
Congestive heart failure	50/1,083 (4.6)	24/335 (7.2)	26/748 (3.5)	0.012^&^
COPD	66/1,083 (6.1)	17/335 (5.1)	49/748 (6.6)	0.423^&^
Chronic kidney disease	45/1,083 (4.2)	20/335 (6.0)	25/748 (3.3)	0.066^&^
Chronic kidney disease requiring RRT	18/1,083 (1.7)	6/335 (1.8)	12/748 (1.6)	1.000^&^
Hematologic cancer	21/1,083 (1.9)	12/335 (3.6)	9/748 (1.2)	0.017^&^
Metastatic cancer	9/1,083 (0.8)	4/335 (1.2)	5/748 (0.7)	0.604^&^
Days from hospital to ICU admission	1 (0-2)	0 (0-2)	1 (0-3)	<0.001^#^
Support at ICU admission, n/total n (%)				
Non-invasive ventilation	581/1,425 (40.8)	69/421 (16.4)	512/1,004 (51.0)	<0.001^&^
Mechanical ventilation	122/1,425 (8.6)	49/421 (11.6)	73/1,004 (7.3)	0.010^&^
Vasopressors	100/1,425 (7.0)	35/421 (8.3)	65/1,004 (6.5)	0.260^&^
Renal replacement therapy	4/1,425 (0.3)	2/421 (0.5)	2/1,004 (0.2)	0.727^&^

Values represent median (IQR) or n/n total (%).

* P values were calculated using; ^#^ Mann-Whitney U test; ^&^ χ^2^ test; ^$^ data available for 827 patients (First Wave: 295 patients, Second Wave: 532 patients); ^‡^ patients were categorized according to their MFI values into Non-frail (MFI=0), Pre-frail (MFI=1-2) or Frail (MFI≥3).

SAPS 3: Simplified Acute Physiology Score 3, scores range from 0 to 217, with higher scores indicating more severe illness and higher risk of death; SOFA Score: Sequential Organ Failure Assessment Score, ranges from 0 to 24, with higher scores indicating more severe organ dysfunction; CCI: Charlson Comorbidity Index, based on a point scoring system (from 0 to 37) for the presence of specific associated diseases and is used for prognosis of lethality; MFI: Modified Frailty Index, values from 1 to 11, scored by assigning 1 point for each frailty components (11 possible comorbidities or *deficits*). COPD: chronic obstructive pulmonary disease; RRT: renal replacement therapy; ICU: intensive care unit.

The median (IQR) number of days from hospital admission to ICU admission was higher in the second wave [1 (0-3) *versus* 0 (0-2); p<0.001] than in the first. On ICU admission, patients admitted during the second wave received NIV more frequently (51.0% *versus* 16.4%; p<0.001) and mechanical ventilation less frequently (7.3% v*ersus* 11.6%; p=0.010) than those admitted during the first wave (Table 1).

### Resource use

During the ICU stay, the second-wave group used more NIV (81.3% *versus* 53.4%; p<0.001) and HFNC (63.2% *versus* 23.0%; p<0.001). The median number of days on MV and the proportion of patients requiring MV, vasopressors, RRT, ECMO, or tracheostomy did not differ between the two waves (Table 2).

**Table 2:** Resource use

Resource	All n=1,427	First wave n=421	Second wave n=1,006	p value*
Support during ICU stay, n/total n (%)				
Non-invasive ventilation	1,043/1,427 (73.1)	225/421 (53.4)	818/1,006 (81.3)	<0.001^&^
Mechanical ventilation	494/1,427 (34.6)	137/421 (32.5)	357/1,006 (35.5)	0.315^&^
Vasopressors	434/1,427 (30.4)	143/421 (34.0)	291/1,006 (28.9)	0.068^&^
High flow nasal cannula	733/1,427 (51.4)	97/421 (23.0)	636/1,006 (63.2)	<0.001^&^
Renal replacement therapy	149/1,427 (10.4)	35/421 (8.3)	114/1,006 (11.3)	0.108^&^
ECMO	20/1,427 (1.4)	6/421 (1.4)	14/1,006 (1.4)	1.000^&^
Tracheostomy, n/total n (%)	92/1,427 (6.4)	26/421 (6.2)	66/1,006 (6.6)	0.879^&^
MV duration (days), median (IQR)	9 (6-23)	9 (5-15)	10 (6-24)	0.170^#^

Values represent median (IQR) or n/total n (%).

* P values were calculated using; ^&^ χ^2^ test; ^#^ Mann-Whitney U test.

ICU: intensive care unit; ECMO: extracorporeal membrane oxygenation; MV: mechanical ventilation.

### Clinical outcomes

**Figure 1:**
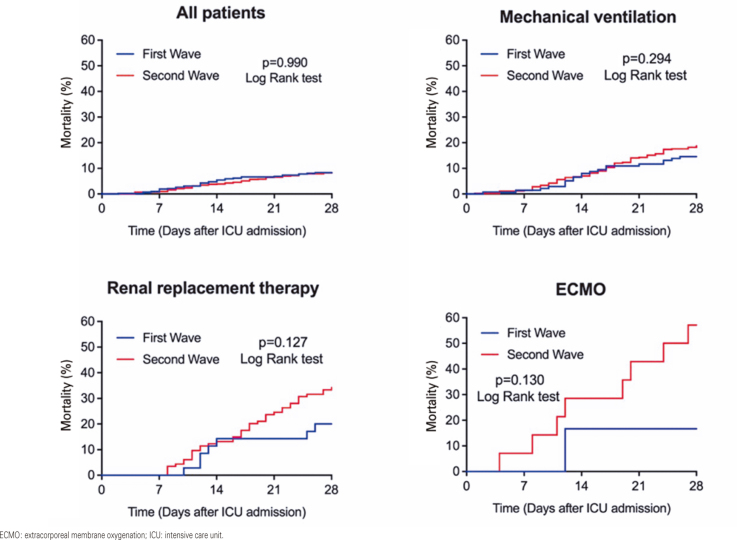
Mortality at day 28 of pooled patients and according to the need of invasive organ support

ECMO: extracorporeal membrane oxygenation; ICU: intensive care unit.

The ICU (11.3% *versus* 10.5%; second and first waves, respectively; p=0.696) and in-hospital mortality (12.3% *versus* 12.1%, second and first waves, respectively; p=0.998) rates did not differ between patients admitted during the second and first waves (Table 3 and Figure 1). Compared with the first wave, the second wave had a longer length of ICU [9 (5-16) days *versus* 8 (4-15) days; p=0.009] and hospital [13 (9-22) days *versus* 12 (8-22) days; p=0.031] stay (Table 3).

**Table 3:** Clinical outcomes stratified according to the use of invasive support

Outcomes	All n=1,427	First wave n=421	Second wave n=1,006	p value*
Destination at hospital discharge, n/total n (%)				0.452^&^
Home	1,220/1,422 (85.8)	358/421 (85.0)	862/1,001 (86.1)	
Home-care	15/1,422 (1.1)	7/421 (1.7)	8/1,001 (0.8)	
Transfer to another hospital	13/1,422 (0.9)	5/421 (1.2)	8/1,001 (0.8)	
Death	174/1,422 (12.2)	51/421 (12.1)	123/1,001 (12.3)	
Palliative care	24/1,422 (1.7)	16/421 (3.8)	8/1,001 (0.8)	<0.001^&^
ICU mortality	158/1,427 (11.1)	44/421 (10.5)	114/1,006 (11.3)	0.696^&^
Hospital mortality	174/1,422 (12.2)	51/421 (12.1)	123/1,001 (12.3)	0.998^&^
ICU LOS (days), median (IQR)	8 (5-16)	8 (4-15)	9 (5-16)	0.009^#^
Hospital LOS (days), median (IQR)	13 (9-22)	12 (8-22)	13 (9-22)	0.031^#^
According to the use of MV				
Patients who received MV, n/total n (%)	494/1,427 (34.6)	137/421 (32.5)	357/1,006 (35.5)	
ICU mortality	128/494 (25.9)	30/137 (21.9)	98/357 (27.5)	0.252^&^
Hospital mortality	138/491 (28.1)	36/137 (26.3)	102/354 (28.8)	0.654^&^
ICU LOS (days), median (IQR)	18 (13-29)^§^	18 (12-30)^§^	18 (13-29)^§^	0.735^#^
Hospital LOS (days), median (IQR)	24 (16-38)^§^	24 (15-41)^§^	24 (16-37)^§^	0.836^#^
Patients who did not receive MV, n/total n (%)	933/1,427 (65.4)	284/421 (67.5)	649/1,006 (64.5)	
ICU mortality	30/933 (3.2)	14/284 (4.9)	16/649 (2.5)	0.078^&^
Hospital mortality	36/931 (3.9)	15/284 (5.3)	21/647 (3.2)	0.194^&^
ICU LOS (days), median (IQR)	6 (3-9)	5 (2-9)	6 (4-9)	0.001^#^
Hospital LOS (days), median (IQR)	11 (8-14)	10 (7-14)	11 (8-14)	0.012^#^
According to the use of RRT				
Patients who received RRT, n/total n (%)	149/1,427 (10.4)	35/421 (8.3)	114/1,006 (11.3)	
ICU mortality	76/149 (51.0)	13/35 (37.1)	63/114 (55.3)	0.092^&^
Hospital mortality	80/149 (53.7)	17/35 (48.6)	63/114 (55.3)	0.617^&^
ICU LOS (days), median (IQR)	26 (15-42)^¶^	25 (12-48)^¶^	27 (16-40)^¶^	0.975^#^
Hospital LOS (days), median (IQR)	34 (18-56)^¶^	36 (17-56)^¶^	33 (18-57)^¶^	0.525^#^
Patients who did not receive RRT, n/total n (%)	1,278/1,427 (89.6)	386/421 (91.7)	892/1,006 (88.7)	
ICU mortality	82/1,278 (6.4)	31/386 (8.0)	51/892 (5.7)	0.154^&^
Hospital mortality	94/1,273 (7.4)	34/386 (8.8)	60/887 (6.8)	0.244^&^
ICU LOS (days), median (IQR)	8 (4-14)	7 (3-13)	8 (5-14)	0.033^#^
Hospital LOS (days), median (IQR)	13 (9-19)	12 (8-19)	13 (9-19)	0.053^#^
According to the use of ECMO				
Patients who received ECMO, n/total n (%)	20/1,427 (1.4)	6/421 (1.4)	14/1,006 (1.4)	
ICU mortality	10/20 (50.0)	1/6 (16.7)	9/14 (64.3)	0.141^$^
Hospital mortality	11/20 (55.0)	2/6 (33.3)	9/14 (64.3)	0.336^$^
ICU LOS (days), median (IQR)	25 (16-45)^‡^	37 (22-55)^†^	25 (12-40)^‡^	0.353^#^
Hospital LOS (days), median (IQR)	31 (16-49)^‡^	49 (27-56)^†^	26 (12-46)^Ψ^	0.153^#^
Patients who did not receive ECMO, n/total n (%)	1,407/1,427 (98.6)	415/421 (98.6)	992/1,006 (98.6)	
ICU mortality	148/1,407 (10.5)	43/415 (10.4)	105/992 (10.6)	0.977^&^
Hospital mortality	163/1,402 (11.6)	49/415 (11.8)	114/987 (11.6)	0.963^&^
ICU LOS (days), median (IQR)	8 (5-16)	8 (4-15)	9 (5-16)	0.007^#^
Hospital LOS (days), median (IQR)	13 (9-21)	12 (8-21)	13 (9-21)	0.020^#^

Values represent median (IQR) or n/total n (%).

* P values were calculated using; ^#^ Mann-Whitney U test; ^&^ χ^2^ test; ^$^ Fisher’s Exact test; ^§^ p<0.001 (Mann-Whitney U test) *versus* patients who did not receive MV; ^¶^ p<0.001 (Mann-Whitney U test) *versus* patients who did not receive RRT; ^‡^ p<0.001 (Mann-Whitney U test) *versus* patients who did not receive ECMO; ^†^ p<0.005 (Mann-Whitney U test) *versus* patients who did not receive ECMO; ^Ψ^ p=0.016 (Mann-Whitney U test) *versus* patients who did not receive ECMO.

ICU: intensive care unit; LOS: length of stay; MV: mechanical ventilation; RRT: renal replacement therapy; ECMO: extracorporeal membrane oxygenation.

Intensive care unit mortality, in-hospital mortality, and ICU and hospital LOS did not differ between patients who received MV, RRT, or ECMO during the first and second waves (Table 3 and Figure 1). Nevertheless, compared to the first wave, the second wave had longer ICU and hospital stays for patients who did not receive MV, RRT, or ECMO (Table 3).

## DISCUSSION

The main findings of this study were that, both the first and second COVID-19 waves had similar mortality rates and the use of invasive resources, despite the second wave patients being younger and less severely ill at ICU admission, according to the SAPS 3 and SOFA Scores. Moreover, during the second wave, patients had longer hospital stays before ICU admission and longer ICU and hospital stays.

Studies from Europe have compared the waves of the COVID-19 pandemic.^(9,10)^ A study from France reported no difference in ICU mortality between the first and second waves. However, they observed a lower proportion of patients requiring invasive mechanical ventilation and a lower rate of thrombotic events in the second wave.^(9)^ In another study from Switzerland, mortality and the need for ICU admission were similar in both waves despite the higher proportion of younger patients admitted to the ICU during the second wave.^(10)^

Unlike developed countries,^(6,8-10)^ Africa^(7)^ and Brazil,^(4,5)^ had a more aggressive second wave than the first wave as the demand for hospital admissions increased, and the proportion of patients requiring advanced respiratory support was higher.

In contrast to other studies conducted in Brazil,^(4,5)^ we did not observe increased ICU and in-hospital mortality rates when the second and first COVID-19 waves were compared. Additionally, the observed mortality in both waves of our cohort was lower than that reported previously.^(4,5)^ The observed lower mortality rate in our study compared to other studies^(4,22,23)^ may be related to ICU characteristics, *i.e*., organizational factors^(24)^ and ICU staffing patterns,^(25)^ and discrepancies in COVID-19 outcomes across the country, which may be explained by social, political, and economic disparities across the regions affecting the availability of ICU beds and the period between disease onset/need for organ support and hospital/ICU admission.

Similar to our study, Contou et al. reported a longer time between hospital and ICU admissions during the second wave than during the first wave.^(9)^ This finding may be explained by the delayed need for endotracheal intubation and MV, potentially related to the early administration of glucocorticoids and increased use of NIV and HFNC during the second wave. Knowledge of evidence-based treatment with glucocorticoids was not available during the first COVID-19 wave. Meanwhile, during the second wave, the benefits of this therapy had already been disseminated and incorporated into clinical practice.^(26)^

We observed increased use of NIV before ICU admission and increased use of NIV and HFNC during ICU stay in the second wave. A recent adaptive randomized controlled trial showed a significantly lower rate of the composite endpoint (tracheal intubation or mortality within 30 days) in patients with COVID-19 with acute hypoxemic respiratory failure randomized to receive continuous positive airway pressure than in patients randomized to receive conventional oxygen therapy.^(27)^ Nevertheless, compared with patients who received conventional oxygen therapy, those randomized to receive HFNC did not exhibit improved outcomes.^(27)^ One may postulate that the increased use of HFNC during the second wave may have affected the timing of endotracheal intubation and the initiation of MV, which has been associated with poor clinical outcomes.^(28)^ Nevertheless, this hypothesis warrants further investigation.

We observed that patients admitted to the ICU during the second wave were younger than patients admitted to the ICU during the first wave [mean difference: 7.3 years; 95%CI: 5.3-9.3 years; p<0.001). The increased hospitalization among younger patients during the second COVID-19 wave may be related to the vaccination campaign in Brazil, where older adults were prioritized to receive the vaccine first.^(4)^ Another possible explanation is related to the behavior of P.1 (gamma) and P.2 variants, which were the prevalent COVID-19 variants during the second wave in Brazil, among younger age groups.^(4,29,30)^ According to a study conducted in European countries, patients infected with the P.1 variant who were younger than 60 years had a higher risk of hospitalization and ICU admission than older patients.^(31)^

A recent meta-analysis of 42 studies and 423,117 patients hospitalized with SARS-CoV-2 infection showed that increased age, male sex, smoking, and the presence of comorbidities such as chronic obstructive pulmonary disease, cardiovascular disease, diabetes, systemic hypertension, obesity, cancer, and acute kidney injury were significantly associated with COVID-19 mortality.^(32)^ Similarly, in our study, we observed that older age and a higher Charlson Comorbidity Index were independently associated with increased COVID-19 mortality.

Our study had several limitations. First, because the study was performed in a single ICU in a private quaternary care hospital in Brazil, the results may not be generalizable to other ICUs in Brazil or outside the country. Second, because there is no standard definition of COVID-19 wave boundaries, we arbitrarily defined the beginning and end of the first and second waves in our study. This may have affected our results and precluded a comparison with other authors. Third, we did not collect detailed data on SARS-CoV-2 variants, which may affect clinical outcomes in patients with severe COVID-19.

Further, we used the SAPS 3 Score to quantify the severity of illness at the time of ICU admission, which may not fully reflect the severity of COVID-19 in our analysis.^(33)^ Also, vaccination against SARS-CoV-2 may have affected clinical outcomes during the second wave. Nevertheless, data on the patients’ vaccination status were not recorded, precluding us from further exploring this hypothesis. Additionally, the small sample size of patients with COVID-19 who received ECMO and the nature of the subgroup analysis precluded us from proposing mechanistic explanations for our findings. Finally, this was an observational, retrospective, single-center study. Therefore, hypothesis generation must be considered.

## CONCLUSION

In the first and second waves, patients with severe COVID-19 exhibited similar mortality rates and need for invasive organ support, despite the patients in the second wave being younger and less severely ill at the time of intensive care unit admission.
